# Micro- and nanoplastics at the vascular interface: experimental insights into endothelial pathways and clinical evidence

**DOI:** 10.1186/s12989-026-00714-4

**Published:** 2026-11-11

**Authors:** Nicole Trippel, Hannes Schiebisch, Fabian Brüstle, Bernhard Schieffer, Karsten Grote

**Affiliations:** Department of Cardiology and Angiology, Philipps-University Marburg, Hans-Meerwein-Straße 2, 35043 Marburg, Germany

**Keywords:** Microplastics, Nanoplastics, Endothelial cells, Endothelium, Cardiovascular disease, Atherosclerosis

## Abstract

Micro- and nanoplastics (MNPs) are increasingly recognized as environmental contaminants with potential implications for cardiovascular health. Their detection in the human circulation and vascular tissues raises the question of how circulating plastic particles interact with the vessel wall. As the primary cellular interface between the circulation and surrounding tissues, the vascular endothelium is uniquely positioned to encounter circulating MNPs. In this Review, we integrate experimental and human evidence on MNP-endothelium interactions and critically examine whether endothelial injury may provide a mechanistic link between circulating MNPs and cardiovascular disease. Experimental studies increasingly support direct effects of MNPs on endothelial homeostasis, including inflammatory and oxidative stress responses, impaired barrier integrity, and alterations in endothelial phenotype and function. Human studies, in contrast, primarily demonstrate circulating and vascular MNP burden and associations with atherosclerotic disease and cardiovascular outcomes, without yet establishing direct endothelial dysfunction or causality. Major differences between experimental exposure conditions and human internal exposure, together with analytical uncertainties in MNP detection, further complicate translation of these findings. Understanding how the endothelium functions as an interface for circulating MNPs, and to what extent it mediates their vascular effects, will be essential for defining the cardiovascular relevance of human MNP exposure.

**Supplementary Information:**

The online version contains supplementary material available at https://doi.org/10.1186/s12989-026-00714-4.

## Clinical trial number

Not applicable.

**Supplementary Information:**

The online version contains supplementary material available at https://doi.org/10.1186/s12989-026-00714-4.

## Micro- and nanoplastics: production, environmental contamination, detection, and potential hazards

The term “plastic” colloquially refers to a broad and chemically diverse group of synthetic polymeric materials. Many conventional plastics are derived from fossil-based petrochemical feedstocks, which are processed into monomers and subsequently polymerized to produce different polymer types. The material is versatile, ranging from less stable disposable items such as polyethylene packaging to highly dimensionally stable polyurethane parts for the automotive industry. The versatility and relatively low production costs of plastics have contributed to their widespread industrial use. Global plastic production has increased substantially since the 1950s. Most of the production now takes place in Asia, mainly in China, while the production rate in Europe has not changed substantially over the last 25 years. Globally, production currently stands at almost 500,000,000 tons per year, of which about half is used for disposable items and only 10% is recycled [42]. Moreover, the recyclability of plastic materials varies considerably among polymer types and products. Microplastics are generally defined as plastic particles ranging from 1 μm to 5 mm, whereas particles smaller than 1 μm are commonly referred to as nanoplastics, although size definitions vary across the literature. In this Review, microplastics and nanoplastics are collectively referred to as micro- and nanoplastics (MNPs).

MNPs enter the environment in various ways. Primary MNPs are produced directly for use in cosmetic products, detergents, and cleaning agents, or are created through wear and tear, e.g., tire abrasion or washing textile clothing. Secondary MNPs, on the other hand, are created by the decomposition and degradation of larger plastic particles (macroplastics) in the environment, e.g., through UV light, saltwater, etc. MNPs are widely distributed in water, soil, and air and can contribute to environmental particulate matter [13]. The composition and abundance of environmental particulate matter are influenced by both natural and anthropogenic sources. Inadequate management and disposal of plastic waste may further contribute to the environmental release and redistribution of plastic-derived particles [25].

In many countries, particularly in urban areas, air-quality monitoring networks routinely measure particulate matter, nitrogen oxides, and ozone. However, conventional air-quality monitoring does not provide polymer-specific characterization of airborne MNPs, particularly at the nanoscale, limiting reliable estimates of inhalational exposure. Estimates of human MNP intake vary widely and remain subject to substantial methodological uncertainty. Exposure may occur through ingestion and inhalation, with dermal exposure considered an additional route [61].

MNPs may also carry plastic-associated chemicals and adsorbed environmental constituents. They contain, for example, plasticizers (phthalates), which are added to plastics during production to make them more flexible. In addition, MNP surfaces may acquire an eco-corona composed of organic substances such as carbohydrates, proteins, DNA, and organic acids, as well as environmental contaminants. MNPs may therefore act as carriers of additional potentially hazardous substances [28].

Early MNP research focused predominantly on the detection and characterization of plastic particles in environmental matrices such as water and soil. In such analyses, polyethylene (PE), polyethylene terephthalate (PET), polypropylene (PP), and polystyrene (PS) are usually among the most frequently detected polymers (e.g., [2]). More recently, experimental studies have increasingly investigated the biological effects of MNPs in cell culture and animal models. In 2021, we demonstrated that PS particles activate endothelial cells and monocytes, are taken up by neutrophils in the blood, and accumulate in the liver, where they cause an inflammatory response [48]. MNP-induced alterations in inflammatory and metabolic pathways have subsequently been reported across multiple cell types, polymer types, and particle sizes [6]. In biopsies from patients or blood, detecting MNPs is significantly more difficult than in soil or water samples, but advances in analytical methods have increasingly enabled their detection in human biological samples. Plastic particles have now been reported in multiple human tissues and body fluids.

## Endothelial cells: morphology and function

Endothelial cells (ECs) form a continuous, highly specialized monolayer that lines the luminal surface of all blood vessels and constitutes the first interface for molecules, cells, or pathogens circulating in the bloodstream [16]. Endothelial dysfunction is associated with a broad spectrum of critical human diseases such as atherosclerosis, hypertension, diabetes mellitus, chronic kidney disease, inflammatory and autoimmune disorders, cancers and neurovascular diseases, highlighting the central role of the endothelium. In fact, ECs and the endothelium are increasingly viewed as an active, distributed organ that integrates biochemical stimuli and hemodynamic forces to control permeability, vascular tone, coagulation balance and immune cell trafficking [4]. The detection of MNPs in the circulation therefore raises the question of how these particles interact with the vascular endothelium and whether such interactions may compromise endothelial homeostasis.

The identity of ECs is established during embryogenesis through vasculogenesis, during which stem and endothelial progenitor cells differentiate and assemble the initial vascular plexus. Subsequent angiogenesis expands, forms a lumen and remodels this network, building an organized vascular system composed of arteries, veins and capillaries. In adult tissues, ECs exhibit a quiescent phenotype under homeostatic conditions. However, they retain a capacity for adaptation that underlines the heterogeneity observed across vascular beds and organs [4]. Structural heterogeneity can be classified into continuous, fenestrated and discontinuous phenotypes, which reflect tissue-specific permeability requirements. Continuous endothelium is therefore predominantly found in organs with strict barrier demands, such as the central nervous system, where dense tight junctions form the basis of the blood-brain barrier (BBB). Fenestrated endothelium is found in the renal glomerulus, facilitating renal filtration. The discontinuous endothelium is found in liver sinusoids, the spleen and bone, permitting extensive molecular exchange [43]. Endothelial phenotypes also vary along the vascular tree. ECs adapt to local mechanical and biochemical conditions, including variations in shear stress and wall tension, which result in distinct morphological and functional characteristics between arterial, venous and capillary segments. These differences are reflected in variable endothelial responsiveness, permeability and biosynthetic activity, contributing to the regulation of vascular tone, coagulation balance and immune response [16].

As the primary cellular interface between blood and vascular tissues, the endothelium determines whether circulating molecules, cells, and particles interact with, traverse, or are retained at the vessel wall. Endothelial barrier function is maintained by the continuous endothelial monolayer, which forms a selectively permeable interface between blood and surrounding tissues. Barrier integrity is regulated by intercellular junctions and controlled transport mechanisms, enabling physiological exchange while preserving vascular stability. Tight junctions seal the continuous endothelium and are particularly prominent in barrier-forming vascular beds, whereas adherens junctions provide a more plastic regulation of permeability in venous and capillary segments. Adherens junctions are formed by vascular endothelial (VE)-cadherin, whose phosphorylation-dependent internalization allows rapid and reversible remodeling of endothelial integrity in response to inflammatory or vasoactive stimuli [10]. In parallel, transcellular transport pathways enable regulated vesicular trafficking and transcytosis of macromolecules, therefore complementing junctional control of endothelial permeability. Dysregulation of these processes leads to pathological hyperpermeability and loss of vascular homeostasis [4].

ECs regulate vascular tone through the release of vasoactive mediators, including endothelial nitric oxide (NO) produced by endothelial nitric oxide synthase (eNOS), prostacyclin and endothelin, thereby coupling local blood flow to tissue demands [4]. While reactive oxygen species (ROS) physiologically act as second messengers in endothelial signaling, excessive ROS production promotes endothelial dysfunction by compromising vascular barrier integrity and reducing nitric oxide bioavailability [44]. At the vascular interface, ECs maintain an antithrombotic surface under physiological conditions but can shift toward procoagulant activity upon injury or activation, including tissue factor expression and von Willebrand factor release, thereby linking endothelial activation to prothrombotic consequences [39].

In response to inflammatory stimuli, endothelial activation promotes leucocyte recruitment and extravasation through multiple coordinated mechanisms, including changes in vascular tone and the regulated expression of adhesion molecules. These processes are mediated by signaling events and transcriptional programs; among them, nuclear factor kappa-light-chain-enhancer of activated B cells (NF-κB) contributes to inflammatory endothelial activation by inducing molecules such as intercellular adhesion molecule-1 (ICAM-1) and vascular cell adhesion molecule-1 (VCAM-1) that support adhesion and transmigration of leucocytes [16]. These mechanisms make endothelial inflammatory activation a particularly relevant response to circulating environmental stressors.

Endothelial plasticity also extends beyond acute responses to long-term remodeling processes. Angiogenesis is a fundamental mechanism of vascular development and tissue growth and is reactivated during tissue repair and tumor progression. Vascular endothelial growth factor (VEGF) acts as a central regulator of angiogenesis by controlling endothelial activation, proliferation, survival, migration and vascular permeability [4, 21]. Under chronic inflammatory or fibrotic conditions, ECs may acquire mesenchymal characteristics through endothelial-to-mesenchymal transition (EndMT), characterized by the loss of endothelial properties and the acquisition of mesenchymal, matrix-producing and contractile phenotypes. EndMT may thereby contribute to fibrosis, vascular remodeling, inflammation, vascular dysfunction and tumor progression [41].

Given their strategic position at the blood-tissue interface and their central role in vascular homeostasis, ECs are uniquely positioned to encounter circulating MNPs and may represent an important target of their vascular effects. While MNPs have increasingly been implicated in cardiovascular pathology, the specific contribution of the endothelium remains incompletely understood. This Review therefore focuses on the vascular endothelium as both a potential site of MNP interaction and a mediator of downstream vascular responses, integrating mechanistic evidence from experimental models with emerging observations from human studies.

## Methods of literature research

**Fig. 1:**
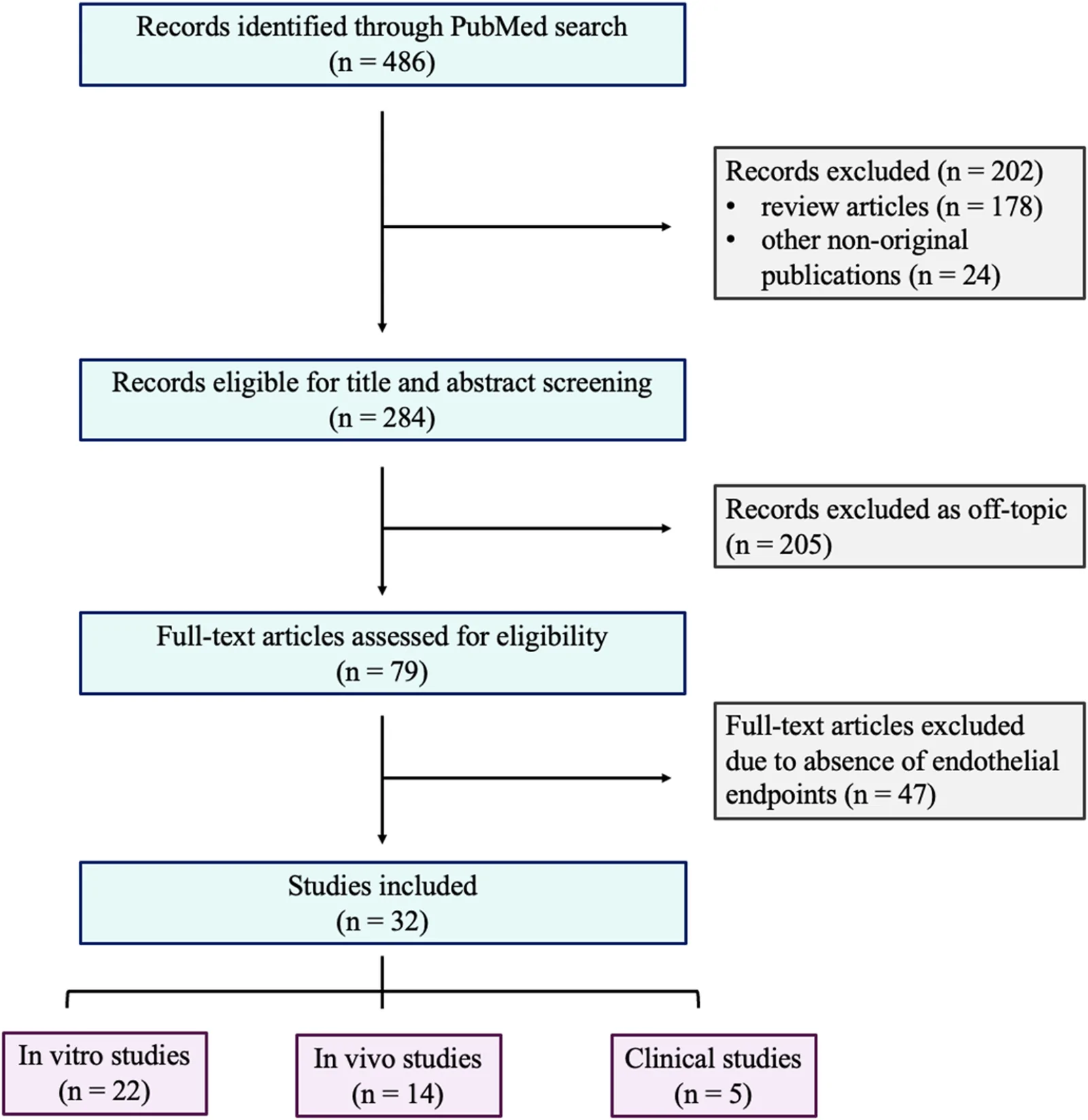
Flow diagram of the literature search and study selection process. A structured PubMed search identified 486 records. The search terms were: (microplastic* OR nanoplastic* OR “micro plastic*” OR “nano plastic*”) AND (endothel* OR vascular OR vasculature OR artery OR arteries OR arterial OR atherosclero* OR atheroma* OR cardiovascular). After exclusion of reviews and other non-original publications, records were screened for relevance based on title and abstract, followed by full-text assessment of potentially eligible studies. Included studies were categorized as in vitro, in vivo, or clinical studies. Categories were not mutually exclusive, as individual studies could contribute to more than one category. Therefore, category-specific counts exceed the number of unique studies included

This overview was compiled from scientific articles published on the effects of MNPs on the structure and function of endothelial cells. Given the biomedical focus of this review on endothelial cell biology and endothelial dysfunction in the context of cardiovascular disease, PubMed was used as the primary literature database. PubMed was searched from database inception through September 6, 2026. The search terms were (microplastic* OR nanoplastic* OR “micro plastic*” OR “nano plastic*”) AND (endothel* OR vascular OR vasculature OR artery OR arteries OR arterial OR atherosclero* OR atheroma* OR cardiovascular), with the search restricted to titles and abstracts. Reviews, meta-analyses, editorials, commentaries, conference abstracts, preprints, and other non-original publication types were excluded. Records were subsequently screened based on title and abstract, followed by full-text assessment of potentially eligible studies. Only peer-reviewed articles published in English were included. Eligible studies investigated the cellular and molecular effects of MNPs on endothelial cells in vitro, endothelial or vascular alterations related to endothelial function in vivo, and associations between MNP exposure or burden and vascular or endothelial health in human clinical or epidemiological studies. Studies without endothelial-related endpoints or vascular outcomes relevant to endothelial dysfunction or employing experimental models unrelated to cardiovascular or vascular biology, were excluded. The study selection process is summarized in Fig. 1. As the database search was restricted to PubMed, individual relevant studies indexed exclusively in other databases may not have been captured.

### Endothelial responses to MNPs: mechanistic in vitro evidence

MNPs have emerged as environmental contaminants capable of altering endothelial cell function. In total, 22 in vitro studies, including two with complementary ex vivo components, have characterized endothelial responses to MNP exposure. Most investigations employed human umbilical vein endothelial cells (HUVECs) as the primary model system. Collectively, these studies indicate that MNP exposure is associated with diverse molecular pathways, including inflammatory activation, oxidative and mitochondrial stress, autophagy-related responses, and disruption of endothelial barrier integrity. This section summarizes these mechanistic axes and highlights key experimental variables relevant for interpreting endothelial responses to MNPs.

### Endothelial activation and inflammatory signaling

Early work by our own group established that murine myocardial microvascular endothelial cells (MyEND cells) mount an inflammatory response to 1 μm carboxylated PS particles (PS-COOH), even at subcytotoxic concentrations (≤ 5.4 µg/mL). Increased expression of *Vcam1* and *Icam1* and enhanced monocyte adhesion point to endothelial activation without detectable loss of cell viability [48]. Beyond adhesion molecule upregulation, MNPs can also trigger NF-κB signaling and necroptotic cell death. Shan et al., reported that 42 nm PS particles activated inflammatory and necroptotic signaling in human brain microvascular endothelial cells (hCMEC/D3). PS exposure increased ROS production and NF-κB activation from 25 µg/mL and tumor necrosis factor-α (TNF-α) release at 100 µg/mL, accompanied by increased receptor-interacting protein kinase 3 (RIPK3), phosphorylated RIPK3 (p-RIPK3), and phosphorylated mixed lineage kinase domain-like protein (p-MLKL) expression. Notably, the reported inflammatory and necroptotic responses occurred at concentrations below those associated with reduced cell viability (200 µg/mL), supporting activation of these pathways under subcytotoxic exposure conditions [45]. In contrast, Lu et al., found no changes in *Vcam1* or *Icam1* expression in HUVECs exposed to 1 μm PS particles at subcytotoxic concentrations between 5 and 25 µg/mL [29]. More recently, Lv et al., showed that 80 nm PS particles increased VCAM-1 and ICAM-1 levels in HUVECs at 100 µg/mL, a concentration exceeding the threshold for reduced cell viability ($\:\ge\:$ 50 µg/mL) [31]. Together, these findings indicate heterogeneous, particle- and concentration-dependent endothelial responses, with inflammatory signaling occurring under subcytotoxic concentrations in some models, while other effects emerge at concentrations associated with reduced cell viability.

### Inflammasome activation and pyroptotic cell death

Huo et al., showed that exposure to 500 nm PS particles activated the NOD-like receptor family pyrin domain-containing 3 (NLRP3) inflammasome-driven pyroptosis pathway in HUVECs. A decrease in cell viability was detectable at 12.5 µg/mL, whereas inflammasome activation became evident from 50 µg/mL onwards, with elevated expression of NLRP3, pro-caspase-1 and its cleaved p20 subunit, as well as gasdermin D (GSDMD) and cleaved GSDMD-N. The secretion of interleukin-1β (IL-1β) and interleukin-18 (IL-18) was likewise increased, consistent with caspase-1 activation. In parallel, PS particle exposure induced an increase in apoptotic cells and significantly elevated lactate dehydrogenase (LDH) release at 100 and 200 µg/mL, indicating membrane damage [17]. Lv et al., similarly demonstrated activation of the NLRP3-caspase-1-GSDMD axis using 80 nm PS particles in HUVECs, with 100 µg/mL increasing NLRP3, apoptosis-associated speck-like protein containing a caspase recruitment domain (ASC), caspase-1, GSDMD and cleaved GSDMD-N, together with higher IL-1β levels [31]. Together, these studies support NLRP3 inflammasome activation and pyroptosis as a response to PS exposure, although the reported effects occurred at concentrations associated with impaired cell viability.

### Oxidative stress, apoptosis and mitochondrial dysfunction

Wang et al., reported that exposure of HUVECs to 500 nm polypropylene (PP) particles activated inflammatory signaling and induced early oxidative stress. Enhanced ROS generation was detectable from 1.5 µg/mL onwards, together with increased secretion of TNF-α and IL-6. LDH release increased from 17.5 µg/mL, whereas endothelial viability declined from 35 µg/mL onwards, accompanied by increased apoptotic and necrotic cell death and altered expression of apoptosis-related genes, including increased B-cell lymphoma 2 (Bcl-2)-associated X protein (Bax), p53 and caspase-3 and decreased Bcl-2. These findings suggest that PP particles elicit inflammatory and oxidative stress at low concentrations below those associated with reduced cell viability, whereas apoptotic and necrotic cell death become evident at higher, cytotoxic concentrations [50]. Yun likewise observed increased apoptosis in human primary retinal microvascular endothelial cells (HRMECs) exposed to 2 μm PS particles. Exposure to 20 µg/mL and higher increased cleaved caspase-3 and Bax while reducing Bcl-2, and was accompanied by inhibition of survival signaling pathways, including reduced phosphorylation of protein kinase B (Akt) and extracellular signal-regulated kinases 1 and 2 (ERK1/2) and reduced cell viability [58].

Further mechanistic insights into MNP toxicity have been obtained from studies examining mitochondrial function. Fu et al., showed that exposure to 50 nm PS particles with different surface functionalizations induced marked oxidative stress in HUVECs. The strongest effects were observed with amine-functionalized PS particles (PS-NH_2_), which, from 10 µg/mL onwards, induced ROS accumulation, mitochondrial membrane depolarization, dysregulation of mitochondrial dynamics (fusion and fission genes), and adenosine triphosphate (ATP) depletion. Notably, reduced cell viability was already observed from 5 µg/mL for PS-NH_2_, indicating that these mitochondrial effects occurred at concentrations associated with cytotoxicity [14]. Chen et al., additionally reported increased ROS production and downregulation of heme oxygenase-1 (HO-1), suggesting impaired antioxidant defense, in the human endothelial cell line EA.hy926 exposed to 2.2–6.5 μm PS particles already at 4 × 10^− 6^ µg/mL. At 0.04 µg/mL, PS exposure was additionally associated with reduced mitochondrial membrane potential and induction of heat shock protein (HSP) 70 and 90, indicative of cellular stress, together with reduced cell viability. Notably, the PS particles used in this study were synthesized in-house rather than purchased commercially [8]. Together, these studies identify oxidative stress as a recurrent endothelial response across different models and particle types, frequently accompanied by mitochondrial dysfunction and apoptotic signaling. However, the concentrations associated with these effects vary substantially between studies, and several mitochondrial and cell-death endpoints were observed at concentrations associated with reduced cell viability, limiting their direct translational interpretation.

### Autophagy and ferroptosis-like stress

Several studies indicate that MNPs can interfere with autophagy and lysosomal function in endothelial cells. Lu et al., reported that exposure of HUVECs to 100 nm PS particles increased autophagosome formation and elevated the autophagy markers microtubule-associated protein 1 light chain 3-II (LC3-II) and beclin-1. Importantly, the authors demonstrated that 100 nm PS particles impaired autophagic flux, whereas 500 nm particles did not induce comparable alterations. Consistent with this, they observed reduced levels of the lysosomal proteins lysosome-associated membrane protein 2 (LAMP2) and cathepsin at concentrations of 5 µg/mL and higher, indicating lysosomal dysfunction. LDH release increased at 25 µg/mL, whereas reduced cell viability was observed only at 50 µg/mL for 500 nm PS particles, indicating that the observed alterations in autophagic flux occurred below the concentration associated with reduced viability [30]. In line with these findings, Lee et al., demonstrated that 500 nm PS particles induced autophagosome accumulation in HUVECs. Treatment increased the LC3-II/LC3-I ratio and intracellular LC3 accumulation, without directly establishing increased autophagic flux. Functionally, PS exposure led to reduced cell viability that was not associated with apoptosis but rather with increased necrotic cell death, as assessed by Annexin V and propidium iodide staining. Importantly, the decrease in cell viability was effectively rescued by pharmacological inhibition of autophagy using 3-methyladenine and by inhibition of phosphoinositide 3-kinase (PI3K) with LY294002, supporting an involvement of autophagy-related processes in PS-induced endothelial cell death [22].

Wei et al., similarly observed increased LC3-II/LC3-I ratios in HUVECs exposed to 30 nm PS-COOH particles, indicating autophagosome accumulation rather than increased autophagic flux [52]. In brain endothelial cells (bEnd.3), Kim et al., demonstrated that exposure to 50 nm PS-NH_2_ particles induced pronounced cellular stress, with increased ROS and intracellular Fe^2+^ levels and reduced glutathione (GSH). Autophagy inhibition, reflected by reduced beclin-1 and LC3-II/LC3-I ratio and increased p62, was observed at 2.5 µg/mL, while elevated ferritin heavy chain 1 (FTH1) was already detectable at lower concentrations. At 5 µg/mL, autophagy activation with rapamycin reversed the increase in Fe^2+^ and FTH1, linking impaired autophagy to iron accumulation. In a complementary hCMEC/D3 model involving PS-NH_2_-exposed erythrocytes, increased Fe^2+^ and ROS levels, lipid peroxidation, and reduced glutathione peroxidase 4 (GPX4) were observed at 1.25 µg/mL, providing additional evidence consistent with ferroptotic stress [20]. Together, these findings indicate particle- and context-dependent disruption of autophagic homeostasis, with one study additionally providing evidence consistent with ferroptosis-like stress.

### Genotoxicity and senescence

Several studies have identified genotoxic effects of MNP exposure in endothelial cells. Martín-Pérez et al., showed that 50 nm PS, PS-NH_2_ and PS-COOH particles induced ROS production and DNA strand breaks in HUVECs at the tested dose of 100 µg/mL, which was also associated with reduced cell viability [33]. Complementing these observations, Martín-Pérez et al., recently performed transcriptomic profiling of HUVECs exposed to 30, 50 and 100 nm carboxylated PS nanoparticles. Gene set enrichment analysis identified coordinated activation of DNA damage and repair pathways together with inflammatory signaling, cholesterol homeostasis and endothelial plasticity. Functional validation confirmed increased DNA strand breaks and intracellular cholesterol accumulation, indicating that MNP exposure induces broad endothelial stress and metabolic responses rather than isolated activation of individual signaling pathways [35]. Janiga-MacNelly et al., likewise reported DNA damage using the human microvascular endothelial cell line HMEC-1, which was exposed to 3–319 μm PS particles at tested doses of 10 µg/mL and 100 µg/mL. Their study used in-house-produced MNP fragments cut from consumer-grade plastic forks and cups, which may contain additives with additional toxic potential [19].

Senescence represents a chronic form of endothelial dysfunction triggered by sustained oxidative stress and DNA damage. Shiwakoti et al., showed that exposure of primary porcine coronary artery endothelial cells (PCAECs) to 25 nm PS particles was associated with cell-cycle arrest, reflected by increased expression of the tumor-suppressor proteins p53, p21 and p16, together with elevated senescence-associated β-galactosidase activity (SA-β-gal) at tested doses of 1 µg/mL and higher. These findings indicate premature endothelial senescence and link MNP exposure to endothelial aging pathways [46]. Complementary findings suggest that MNP-induced endothelial senescence is mechanistically linked to sodium-glucose co-transporter 2 (SGLT2). In PCAECs, MNP exposure upregulated SGLT2 expression and promoted senescence, whereas pharmacological SGLT2 inhibition attenuated senescence markers and improved endothelial function [12]. Together, these findings suggest that genotoxic stress may contribute to premature endothelial senescence, although evidence for this long-term response remains limited and several reported effects extend into concentration ranges associated with reduced cell viability.

### Endothelial barrier integrity and junctional pathways

Another major mechanistic axis involves junctional signaling and endothelial barrier integrity. Lee et al., provided some of the earliest evidence that MNPs impair endothelial barrier-related functions. Using 500, 1000 and 5000 nm PS particles in HUVECs, they found that only 500 nm particles produced functional impairment, reducing wound healing and cell migration at concentrations of 20 µg/mL and above. These effects were accompanied by suppression of VEGF-dependent signaling pathways involved in endothelial migration and proliferation [22]. Wei et al., later demonstrated that MNP-induced barrier disruption strongly depends on polymer chemistry and surface charge. In HUVECs, 30 nm PS-COOH and carboxylated polymethyl methacrylate particles (PMMA-COOH) increased endothelial permeability at 0.05 mg/mL and caused gap formation accompanied by disrupted VE-cadherin structure. PS-COOH additionally promoted VE-cadherin phosphorylation at positions Y658 and Y731, supporting junctional destabilization. Molecular dynamics simulations indicated that PS-COOH and PMMA-COOH bind to the extracellular cadherin (EC1) domain of VE-cadherin and weaken homophilic dimerization, which may facilitate junction opening under mechanical stress. In contrast, PS-NH_2_ particles did not induce leakiness or gap formation at matched doses. Wei et al., further confirmed increased leakiness ex vivo in rabbit and swine arteries after exposure to 30 nm PS-COOH at 0.05–5 mg/mL [52]. In contrast to the absent effects of PS-NH_2_ reported by Wei et al., in HUVECs, Kim et al., observed pronounced tight-junction impairment in bEnd.3 and hCMEC/D3 cells. Exposure to positively charged 50 nm PS-NH_2_ particles reduced the expression of the junction proteins Zonula Occludens-1 (ZO-1), Occludin and Claudin-5 and lowered transendothelial electrical resistance (TEER) at concentrations between 1.25 and 2.5 µg/mL [20]. Shan et al., similarly observed reduced TEER and Occludin downregulation in hCMEC/D3 cells exposed to 42 nm PS particles, with effects detectable from 50 µg/mL [45]. Further impairments of endothelial repair capacity have been described. Wang et al., showed that 500 nm PP particles impaired experimental wound closure in HUVECs, with effects detectable from 1.5 µg/mL, and reduced ZO-1 expression at higher doses (≥ 210 µg/mL) [50]. Lv et al., also observed impaired wound healing in HUVECs exposed to 80 nm PS particles at 100 µg/mL, a concentration associated with reduced cell viability [31]. More recently, Huo et al., demonstrated that PET MNPs disrupted endothelial glycocalyx homeostasis and junctional integrity in human aortic endothelial cells (HAECs), characterized by reduced syndecan-1 (SDC1) expression, glycocalyx loss, and disrupted β-catenin-positive intercellular junctions. These effects were linked to activation of endoplasmic reticulum (ER) stress and increased ROS production, while pharmacological inhibition of oxidative and ER stress partially restored glycocalyx and junctional integrity, supporting a mechanistic role of the ER stress-ROS axis in PET-induced endothelial barrier dysfunction [18]. Together, these studies identify endothelial barrier disruption as a recurrent response to MNP exposure, with effects strongly influenced by particle physicochemical properties and endothelial cell type.

### Angiogenesis and endothelial-dependent relaxation

Another important function of endothelial cells is angiogenesis. Lee et al., first reported that 500 nm PS particles impaired tube formation in HUVECs, with effects observed from 20 µg/mL, whereas 1000 nm PS particles required higher doses (≥ 80 µg/mL) and 5000 nm particles showed no detectable effect. Importantly, these angiogenic impairments occurred under conditions in which short-term cell viability remained unaffected, indicating that the reduced tube formation was not a consequence of cytotoxicity [22]. Yun subsequently demonstrated that 2 μm PS particles inhibited VEGF-induced angiogenic signaling in human retinal microvascular endothelial cells (HRMECs) by suppressing Akt and ERK1/2 phosphorylation at cytotoxic doses of 20 µg/mL and higher. This led to impaired migration, proliferation and tube formation, all of which were restored by pharmacological activation of Akt or ERK using SC79 (Akt activator) and ceramide C6 (ERK activator) [58]. In contrast, Martín-Pérez et al., observed distinct size-dependent effects of carboxylated PS particles on endothelial migration and angiogenesis. In HUVECs, 30 nm particles accelerated wound closure but reduced the covered area in tube formation assays. Conversely, 50 and 100 nm particles increased covered area and total tube length, indicating increased tube formation. These findings were accompanied by transcriptomic alterations in endothelial plasticity-related pathways, highlighting the strong size dependence of endothelial responses to plastic particles [35]. Consistent with the possibility that MNPs may also promote angiogenesis-related responses, Basini et al., found that exposure of bovine aortic endothelial cells (AOECs) to 100 nm PS particles increased VEGF secretion at doses of 5 µg/mL and higher, without affecting cell proliferation. The authors did not assess whether the highest tested dose of 75 µg/mL influenced cell viability [5]. Together, these findings indicate that MNPs do not uniformly suppress angiogenic processes but can induce divergent responses depending on particle size, dose and endothelial subtype.

A further important function of endothelial cells, endothelium-dependent relaxation, can also be impaired by exposure to MNPs. Shiwakoti et al., demonstrated that 25 nm PS particles reduced bradykinin-induced vasorelaxation in ex vivo porcine coronary arteries at 1 µg/mL. This was accompanied by decreased eNOS expression and reduced NO production, whereas smooth muscle contractile responses remained unaffected, indicating selective impairment of endothelium-dependent relaxation [46]. Together, these findings demonstrate that MNP exposure can alter both angiogenic responses and endothelium-dependent vascular function.

### Endothelial-to-mesenchymal transition

Yang et al., provided evidence that prolonged MNP exposure may induce EndMT. Using 5 μm PS particles, the authors observed that exposure at concentrations of 1 µg/mL and higher reduced the endothelial marker CD31 and increased the expression of mesenchymal markers including α-smooth muscle actin (α-SMA), Vimentin, Snail1 and Twist1. Mechanistically, these changes were associated with activation of the bone morphogenetic protein 2 (BMP-2)-SMAD1/5/8 signaling pathway, suggesting that MNPs can engage BMP-dependent transcriptional programs that promote EndMT [55]. As cytotoxicity was not assessed in this study, it remains unclear whether these phenotypic changes occurred independently of potential effects on cell viability. Although evidence is currently limited to a single study, these findings suggest that prolonged MNP exposure may promote persistent endothelial phenotypic changes relevant to vascular remodeling.

**Fig. 2:**
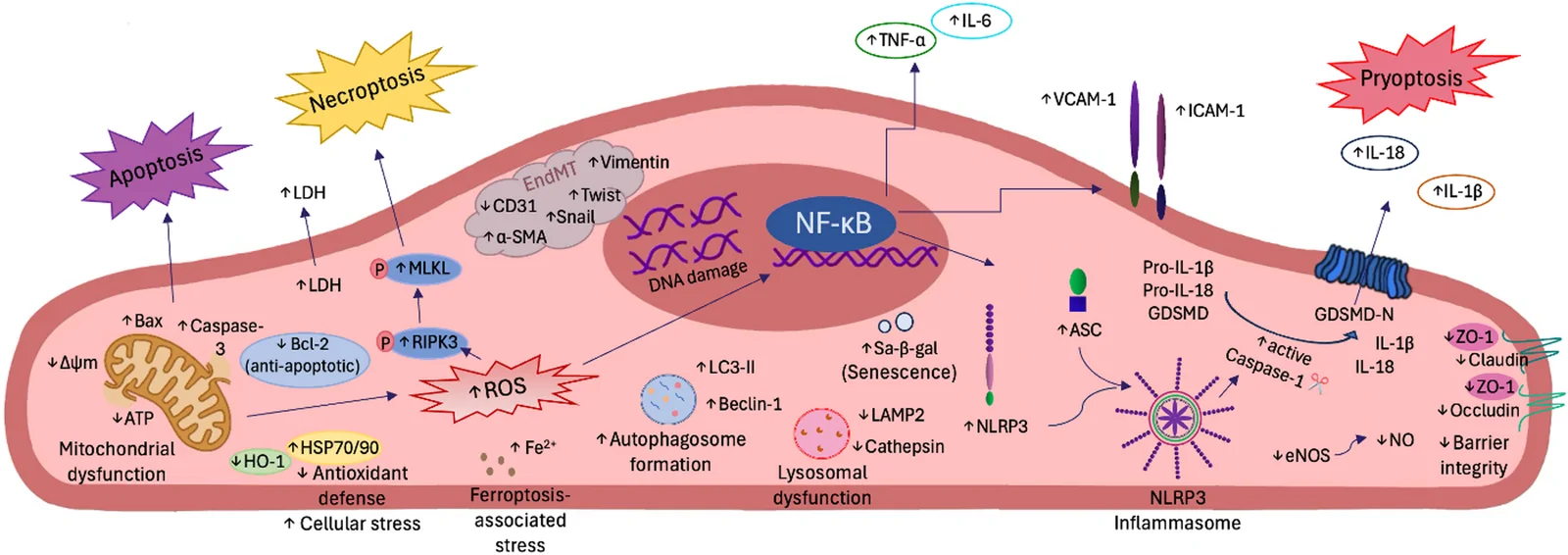
MNP-associated stress and inflammatory pathways in endothelial cells. The schematic summarizes experimentally reported molecular and cellular alterations associated with MNP exposure, including mitochondrial dysfunction and oxidative stress, DNA damage, autophagy-lysosome alterations, and apoptotic, necroptotic, and inflammasome-dependent pyroptotic signaling, as well as ferroptosis-associated stress. In parallel, NF-κB-associated inflammatory signaling and endothelial activation are shown alongside increased EndMT-related marker expression and impairment of endothelial function, including reduced eNOS/NO bioavailability and decreased barrier integrity. The depicted alterations summarize reported experimental findings and do not imply an equivalent level of mechanistic evidence for all pathways. Arrows indicate reported increase or decrease in expression or activity

Collectively, current in vitro evidence shows that MNPs affect endothelial homeostasis through multiple, partly intersecting mechanisms, including inflammatory activation, oxidative and mitochondrial stress, disruption of junctional integrity, altered autophagic homeostasis, angiogenic alterations and phenotypic reprogramming. These responses vary substantially with particle size, surface chemistry, concentration and endothelial model. Oxidative stress, inflammatory activation and endothelial barrier dysfunction have been reported across multiple endothelial models, whereas evidence for pyroptosis, ferroptosis-like signaling, cellular senescence and EndMT remains comparatively limited. Overall, the substantial heterogeneity in particle characteristics, exposure conditions and experimental models complicates direct comparison across studies. All in vitro findings are summarized in Table 1 and illustrated schematically in Fig. 2.

**Table 1:** Summary of endothelial-specific effects of MNPs in vitro

Mechanistic Category	Endothelial cell model	Particle size and type	Relevant concentrations	Main findings	Cytotoxicity		Reference
Endothelial activation & inflammation	MyEnd	1 μm PS-COOH	5.4 µg/mL	Upregulation of adhesion molecules (*Vcam1*, *Icam1*), increased monocyte adhesion	No reduced viability up to 5.4 µg/mL	Vlacil et al., [48]	
	HUVEC	1 μm PS	5–25 µg/mL	No induction of adhesion molecules or inflammatory activation	No reduced viability at 5–25 µg/mL	Lu et al., [29]	
	HUVEC	80 nm PS	100 µg/mL	Upregulation of adhesion molecules (VCAM-1, ICAM-1)	Reduced viability $\:\ge\:$ 50 µg/mL	Lv et al., [31]	
NF-κB signaling & necroptosis	hCMEC/D3	42 nm PS	$\:\ge\:$ 25 µg/mL	Increased ROS and TNF-α release, NF-κB activation and induced necroptotic signaling (↑ RIPK3, p-MLKL)	Reduced viability at 200 µg/mL	Shan et al., [45]	
Inflammasome & pyroptosis	HUVEC	500 nm PS	$\:\ge\:$ 50 µg/mL	Activation of the NLRP3-caspase-1-GSDMD axis with IL-1β release and LDH increase	Reduced viability $\:\ge\:$ 12.5 µg/mL	Huo et al., [17]	
	HUVEC	80 nm PS	100 µg/mL	Activation of the NLRP3-ASC-caspase-1-GSDMD pathway with increased IL-1β	Reduced viability $\:\ge\:$ 50 µg/mL	Lv et al., [31]	
Oxidative stress & apoptosis	HUVEC	500 nm PP	1.5–210 µg/mL	Enhanced ROS accumulation, induced apoptotic signaling (↑ Bax/Bcl-2 ratio, caspase-3)	Reduced viability $\:\ge\:$ 35 µg/mL	Wang et al., [50]	
	HRMEC	2 μm PS	20–40 µg/mL	Induced apoptotic signaling (↑ Bax, ↓ Bcl-2, ↑ caspase-3), suppression of AKT/ERK survival signaling	Reduced viability $\:\ge\:$ 20 µg/mL	Yun, [58]	
Oxidative and mitochondrial stress	HUVEC	50 nm PS-NH_2_	10–25 µg/mL	Enhanced ROS accumulation, mitochondrial membrane depolarization, ATP depletion	Reduced viability $\:\ge\:$ 5 µg/mL	Fu et al., [14]	
	Ea.hy926	2.2–6.5 μm PS	4 × 10^− 6^– 4 × 10^− 2^ µg/mL	Enhanced ROS accumulation, mitochondrial membrane depolarization, HO-1 downregulation, induction of heat shock proteins (HSP70/90)	Reduced viability $\:\ge\:$ 0.04 µg/mL	Chen et al., [8]	
Autophagy & lysosomal dysfunction	HUVEC	100 nm PS 500 nm PS	5–25 µg/mL	Autophagosome accumulation (↑ LC3-II, beclin-1), impaired lysosomal flux, reduced lysosomal protein levels (↓ LAMP2, cathepsin), effects induced only by 100 nm PS	Reduced viability at 50 µg/mL	Lu et al., [30]	
Autophagy-related alterations	HUVEC	500 nm, 1 μm, 5 μm PS	20 µg/mL	Increased necrotic cell death Autophagosome accumulation (Increased LC3-II/LC3-I ratio) Rescue of cell viability by autophagy and PI3K inhibition effects induced only by 500 nm PS	Reduced viability $\:\ge\:$ 20 µg/mL	Lee et al., [22]	
	HUVEC	30 nm PS-COOH 30 nm PS-NH_2_ 30 nm PMMA-COOH	0.05–0.5 mg/mL	Autophagosome accumulation (Increased LC3-II/LC3-I ratio), effects induced only by 30 nm PS-COOH	Reduced viability from 0.05 mg/mL (time-dependent; PS-COOH)	Wei et al., [52]	
Ferroptosis-like stress	bEND.3; hCMEC/D3	50 nm, 100 nm, 1000 nm PS-NH_2_	0.5–5 µg/mL	Iron accumulation, oxidative stress and autophagy inhibition; lipid peroxidation and reduced GPX4 consistent with ferroptosis-like stress	Reduced viability at 5 µg/mL	Kim et al., [20]	
Genotoxicity	HUVEC	50 nm PS 50 nm PS-COOH 50 nm PS-NH_2_	100 µg/mL	Increased ROS production, DNA damage (strand breaks), intracellular vesicle accumulation	Reduced viability at 100 µg/mL	Martín-Pérez et al., [33]	
	HMEC-1	2.99–319 μm PS fragments from plastic forks and cups	10 µg/mL 100 µg/mL	DNA damage (strand breaks)	Reduced viability at 100 µg/mL	Janiga-MacNelly et al., [19]	
Genotoxicity & metabolic alterations	HUVEC	30 nm, 50 nm, 100 nm PS-COOH	100 µg/mL	Transcriptomic alterations in DNA damage/repair, cholesterol homeostasis and endothelial plasticity	Reduced viability at 100 µg/mL	Martín-Pérez et al., [35]	
Senescence	PCAEC	25 nm PS	1–10 µg/mL	Increased SA-β-gal activity, upregulation of p53/p21/p16 (cell cycle arrest-associated markers)	Reduced viability $\:\ge\:$ 5 µg/mL	Shiwakoti et al., [46]	
	PCAEC	20–25 nm PS	1–10 µg/mL	Increased SGLT2 expression increased SA-β-gal activity, upregulation of p53/p21 SGLT2 inhibition (Enavogliflozin) attenuates senescence and improves endothelial function	Reduced viability $\:\ge\:$ 5 µg/mL	Dhakal et al., [12]	
Endothelial barrier integrity, junctional and angiogenic signaling	HUVEC	500 nm, 1 μm, 5 μm PS	20–80 µg/mL	Reduced tube formation, impaired wound closure and cell migration, repression of VEGF-dependent angiogenetic signaling, effects induced only by 500 nm PS	Reduced viability $\:\ge\:$ 20 µg/mL (500 nm PS)	Lee et al., [22]	
	HUVEC	30 nm PS-COOH 30 nm PS-NH_2_ 30 nm PMMA-COOH	0.05–0.5 mg/mL	Enhanced endothelial permeability and gap formation, Src-dependent VE-cadherin phosphorylation at Y658/Y731; direct binding of PS-COOH and PMMA-COOH to VE-cadherin EC1 domain with reduced homophilic dimer stability; junctional effects only induced by PS-COOH and PMMA-COOH	Reduced viability from 0.05 mg/mL (time-dependent; PS-COOH)	Wei et al., [52]	
	Ex vivo: rabbit & swine vessels	30 nm PS-COOH	0.05–5 mg/mL	Enhanced vascular endothelial leakiness	N/A	Wei et al., [52]	
	bEND.3 hCMEC/D3	50 nm PS – NH_2_	1.25–2.5 µg/mL	Induced endothelial leakiness, decreased TEER, Tight junction disruption (↓ ZO-1, Occludin, Claudin-5)	Reduced viability at 5 µg/mL	Kim et al., [20]	
	hCMEC/D3	42 nm PS	50–100 µg/mL	Decreased TEER, Occludin downregulation	Reduced viability at 200 µg/mL	Shan et al., [45]	
	HUVEC	500 nm PP	1.5–210 µg/mL	impaired wound healing, impaired chemotactic cell migration Reduced ZO-1 expression	Reduced viability $\:\ge\:$ 35 µg/mL	Wang et al., [50]	
	HUVEC	80 nm PS	100 µg/mL	Impaired wound healing	Reduced viability $\:\ge\:$ 50 µg/mL	Lv et al., [31]	
	HRMEC	2 μm PS	20–40 µg/mL	Reduced tube formation, impaired cell migration, repression of VEGF-dependent angiogenetic signaling	Reduced viability $\:\ge\:$ 20 µg/mL	Yun, [58]	
	HUVEC	200 nm PS, PLA, PTFE, PET	25 µg/mL	Impaired endothelial migration induced by PTFE and PET; no effect of PS or PLA	No reduced viability up to 25 µg/mL	Martín-Pérez et al., [34]	
	HUVEC	30 nm, 50 nm, 100 nm PS-COOH	100 µg/mL	Size-dependent alterations in migration and angiogenesis, with decreased migration but increased tube formation at larger particle sizes	No reduced viability up to 200 µg/mL	Martín-Pérez et al., [35]	
	HAEC	1–10 μm PET	200 and 500 µg/mL	Glycocalyx loss and junctional disruption associated with ER stress, ROS production and inflammatory activation	No significant reduction in viability reported	Huo et al., [18]	
Angiogenetic stimulation	Aortic endothelial cells	100 nm PS	5–75 µg/mL	Increased VEGF secretion	Not assessed	Basini et al., [5]	
Endothelial vasoactive signaling	Ex vivo: porcine coronary artery	25 nm PS	1 µg/mL	Impaired bradykinin-induced endothelium-dependent relaxation, decreased eNOS expression, reduced NO production	N/A	Shiwakoti et al., [46]	
EndMT	HUVEC	5 μm PS	1–10 µg/mL	Decreased CD31 expression, increased mesenchymal markers (α-SMA, Vimentin, Snail and Twist) via BMP-2–SMAD1/5/8 signaling	Not assessed	Yang et al., [55]	

### In vivo endothelial and vascular effects of MNPs

To date, 14 in vivo studies have investigated endothelial and vascular effects of MNP exposure, using models ranging from zebrafish embryos to wild-type rodents and established genetically modified mouse models of atherosclerosis, including apolipoprotein E-deficient (ApoE^‒/‒^) and low-density lipoprotein receptor-deficient (LDLR^‒/‒^) mice under high-fat diet (HFD) conditions. Across these models, MNP exposure has been associated with oxidative stress, inflammatory activation, EndMT, prothrombotic signaling, impaired angiogenesis, and endothelial barrier dysfunction.

### Effects of MNPs on vascular development in vivo

Using Tg(kdrl: eGFP) zebrafish embryos, which express green fluorescent protein in endothelial cells, Chen et al., exposed embryos starting 2 h post-fertilization (hpf) to 20 nm plastic particles at exposure concentrations of 2, 5, or 8 mg/L in E3 medium. Notably, the chemical composition and surface characteristics of the applied plastic particles were not further specified. The authors observed a dose-dependent impairment of caudal vein plexus (CVP) development, with reduced vascular sprouting, fewer loop structures, and decreased ventral extension already at 30 hpf, and severe CVP malformations at 8 mg/L at 48 hpf. These defects persisted after transferring to fresh E3 medium, indicating persistent vascular developmental impairment at concentrations of 5 and 8 mg/L. Transcriptomic and single-cell RNA sequencing analyses revealed alterations in endothelial redox-related pathways and extracellular matrix organization. Co-exposure to hydrogen peroxide (H_2_O_2_) further aggravated CVP damage, whereas antioxidant co-treatment with N-acetylcysteine (NAC) partially restored vascular development, supporting oxidative stress as a mediator of particle-induced developmental vascular toxicity in this developmental model [7].

### Endothelial injury, remodeling, and barrier disruption in wild-type rodents

Previous work from our group demonstrated acute endothelial activation following a single intravenous injection of 2.5 mg of 1 μm PS-COOH in wild-type C57BL/6 N mice. Three hours after injection, aortic tissue showed increased expression of *Il1b* and *Vcam1*, indicating rapid vascular inflammatory activation in response to circulating PS particles [48]. Complementing these inflammatory activation responses, Wei et al., demonstrated that 30 nm PS-COOH particles induced pronounced endothelial barrier disruption in vivo. Male Swiss mice received a single intravenous injection of Evans blue dye containing PS particles at doses of 1.5, 15, or 30 mg/kg body weight (bw), while control animals received Evans blue alone. After 24 h, mice in the PS-exposed groups exhibited dose-dependent Evans blue extravasation mainly in the brain, liver, spleen, and lungs, indicating widespread vascular leakage. In a subcutaneous vascular leakage model, PS particles (30 mg/kg bw) likewise induced marked dermal Evans blue extravasation [52]. Endothelial responses to repeated intravascular MNP exposure were further investigated by Wang et al., in male BALB/c mice. Animals received 80 nm pristine PS at 5–20 mg/kg bw or surface-modified PS-NH_2_ and PS-COOH at 0.05–5 mg/kg bw by caudal vein injection every other day for 14 days. Aortic histology revealed poor nuclear homogeneity, dose-dependent inflammatory cell infiltration, wall thickening, and structural disorganization. Ultrastructural analysis revealed organelle disintegration in PS-treated endothelium. At higher doses, endothelial cells became pyknotic and exfoliated. PS-NH_2_ exposure induced prominent ER vacuolization and nuclear pyknosis, while PS-COOH caused progressive nuclear pyknosis, cellular deformation, vacuolization, and endothelial cell abscission at the highest dose. Systemic and vascular inflammation was reflected by a dose-dependent increase in serum IL-6 and C-C motif chemokine ligand-2 (CCL2) across all PS particles. In parallel, aortic expression of Janus kinase 1 (JAK1) and signal transducer and activator of transcription 3 (STAT3) was increased, consistent with activation of cytokine-associated inflammatory signaling within the vessel wall. Coagulation parameters were also altered, with shortened prothrombin time (PT) and activated partial thromboplastin time (aPTT), as well as elevated plasma fibrinogen. These functional changes were accompanied by increased aortic expression of tissue factor (TF), protease-activated receptor-1 (PAR1), and fibrinogen, indicating a shift toward a prothrombotic vascular phenotype [51].

While intravenous administration does not reflect conventional environmental exposure through ingestion or inhalation and likely maximizes direct endothelial contact, these models provide proof-of-principle evidence of vascular responses to circulating MNPs. Direct intravascular exposure may additionally be relevant in specific clinical settings involving plastic-containing medical devices, as discussed below. Subsequent experimental studies increasingly employed oral exposure models.

Using a daily oral gavage approach, Shan et al., treated male C57BL/6J mice with 42 nm PS particles at 0.5–50 mg/kg bw for 7 days and detected administered particles in brain tissue following gastrointestinal exposure. However, detection in brain tissue does not by itself distinguish parenchymal translocation from retention within the cerebral vasculature or endothelium. Concomitantly, expression of platelet endothelial cell adhesion molecule-1 (PECAM-1) in brain vessels was reduced, and fluorescent tracer experiments demonstrated increased BBB permeability, indicating that small PS particles can compromise blood-brain barrier integrity in vivo [45].

In male Wistar rats, Huo et al., exposed animals by daily oral gavage to 500 nm PS particles at doses of 0.5, 5, or 50 mg/kg bw for 90 days, followed by structural and molecular analyses of aortic vessels. Histopathological evaluation revealed dose-dependent endothelial disorganization, inflammatory cell infiltration, and vascular wall thickening at doses of 0.5 mg/kg bw and higher. At the molecular level, PS exposure induced pronounced oxidative stress in aortic tissue, as evidenced by increased malondialdehyde (MDA) levels and concomitant depletion of superoxide dismutase (SOD) and glutathione (GSH). Inflammatory cytokine expression showed a dose-dependent pattern, with increased levels of the anti-inflammatory cytokine interleukin-10 (IL-10) at lower doses (0.5–5 mg/kg bw), whereas the pro-inflammatory cytokine IL-6 was significantly upregulated only at the highest dose (50 mg/kg bw). In addition to cytokine alterations, PS exposure was associated with increased expression of key components of the NLRP3 inflammasome pathway, including NLRP3, caspase-1, and GSDMD in aortic tissue. These molecular alterations coincided with structural endothelial injury and vascular remodeling [17].

Evidence from a chronic drinking-water exposure model further supports direct interactions of MNPs with the aortic endothelium. Huo et al., exposed male Sprague-Dawley rats to 1–10 μm PET particles via drinking water at concentrations of 1, 10, or 100 mg/L for 8 weeks. Transmission electron microscopy demonstrated PET particle deposition within aortic endothelial cells, whereas particles were not detected within the elastic fiber layer. Exposure altered aortic elastic fiber morphology without significantly affecting aortic wall thickness. At 100 mg/L, reduced SDC1 expression and decreased staining of endothelial glycocalyx components indicated impaired glycocalyx integrity, accompanied by increased ICAM-1 expression consistent with endothelial activation. Transcriptomic analyses further linked impaired glycocalyx homeostasis to alterations in extracellular matrix-related pathways and elastic fiber morphology. These findings associate endothelial PET particle deposition and glycocalyx disruption with early structural remodeling of the aortic wall [18].

Functional consequences of oral PS exposure were further demonstrated by Lv et al., in male C57BL/6J mice treated with 80 nm PS particles by daily oral gavage at 80 mg/kg bw for 12 weeks. PS exposure resulted in increased arterial stiffness, as reflected by elevated ultrasound-derived pulse wave velocity, along with increased aortic wall thickness, enhanced fibrosis and collagen deposition, and reduced expression of the endothelial marker CD31. In parallel, the vascular adhesion molecules ICAM-1 and VCAM-1 were upregulated in arterial tissue, consistent with vascular inflammatory activation and arterial wall remodeling [31].

Beyond large-vessel remodeling, Xu et al., extended these observations to the renal microvasculature. Male C57BL/6 mice were exposed to 1 μm PS particles via drinking water (10 mg/L) for 18 weeks, either under normal diet or HFD conditions. Single-cell RNA sequencing of kidney tissue identified three major endothelial subtypes: arterial, capillary, and glomerular endothelial cells. PS exposure increased the proportion of glomerular endothelial cells while reducing arterial endothelial cells, whereas combined exposure to PS and HFD further increased capillary endothelial cells. At the signaling level, PS exposure was associated with activation of the PI3K-Akt pathway, MAPK pathway, and the interleukin-17 (IL-17) pathway, indicating inflammatory and stress-related endothelial signaling. Together, these findings indicate MNP-associated changes in renal endothelial composition and signaling that may be further modified by dietary lipid stress [54].

### Endothelial and vascular effects in atherosclerosis models

In ApoE^‒/‒^ mice, Yang et al., investigated the impact of MNP exposure on atherosclerotic plaque development and vascular alterations, including endothelial-associated molecular changes. Six-week-old male ApoE^‒/‒^ mice fed a HFD received drinking water containing 5 μm PS particles at 1–10 µg/mL for 12 weeks. PS exposure aggravated atherosclerosis, with increased plaque burden and lipid accumulation in aortic lesions. In aortic tissue, mRNA expression of *Icam1*,* Vcam1*,* Ccl2*, and matrix metalloproteinase-9 (*Mmp9*) was upregulated, whereas expression of *Nos3* (encoding eNOS) was reduced, consistent with endothelial activation and reduced endothelial homeostatic signaling. Aortic oxidative stress was increased, with reduced antioxidant capacity and enhanced ROS accumulation. Concomitantly, PS exposure promoted EndMT in aortic tissue, as reflected by reduced VE-cadherin expression together with increased protein levels of Vimentin, α-SMA, and the EndMT-associated transcription factors Snail1 and Twist1. These changes were accompanied by activation of BMP-SMAD1/5/8 signaling [55].

Other studies in atherosclerosis-prone mice predominantly focused on vascular remodeling and lesion-level outcomes. Wen et al., reported accelerated atherosclerotic lesion development in ApoE^‒/‒^ mice exposed to 100 nm PS particles for 3 months, with increased plaque formation and lipid deposition, reduced local blood flow velocity, and increased plaque collagen content [53]. Long-term oral exposure of ApoE^‒/‒^ mice to 50 nm PS particles for 19 weeks likewise aggravated vascular remodeling and atherosclerosis, with increased arterial stiffness, intima-media thickness, plaque burden, lipid deposition, and collagen accumulation [49]. In a related study, ApoE^‒/‒^ mice exposed to 500 nm PS particles via drinking water for 20 weeks showed increased lipid accumulation in the heart valve area. RNA sequencing of aortic lesions further revealed differential gene expression predominantly involving immune cell activation, cell adhesion, and inflammatory pathways [60].

In a study directly comparing both sexes under identical exposure conditions, daily oral administration of 1.1 μm PS particles at 10 mg/kg bw for 9 weeks markedly aggravated atherosclerosis in male LDLR^‒/‒^ mice, whereas female mice remained largely unaffected. In males, lesion areas increased by 63% in the aortic root and more than sevenfold in the brachiocephalic artery, accompanied by increased plaque macrophage content without changes in SMC abundance. Single-cell RNA sequencing further revealed remodeling of endothelial cell subpopulations in exposed males. The proportion of an endothelial cluster characterized by *Pecam1* and bone morphogenetic protein 6 (*Bmp6*) expression decreased, whereas a cluster characterized by fatty acid-binding protein 4 (*Fabp4*) and cluster of differentiation 36 (*Cd36*) expression and associated with lipid handling and inflammatory activation expanded. Pathway analysis further implicated alterations in cell-cell adhesion, endothelial proliferation, fatty acid transport, and lipid homeostasis [24]. Notably, most rodent studies included in this review were conducted exclusively in male animals. The marked sex-specific response observed by Lin et al., suggests that sex may modify vascular susceptibility to MNP exposure. However, the limited representation of female animals currently precludes systematic conclusions regarding sex-dependent effects.

Collectively, in vivo studies provide evidence of both direct endothelial alterations and broader vascular responses to MNP exposure. Evidence directly involving the endothelium includes barrier disruption, glycocalyx impairment, endothelial activation, and altered endothelial phenotypes, whereas several studies, particularly in atherosclerosis-prone models, primarily report vascular remodeling and lesion-level outcomes. In atherosclerosis-prone models, MNP exposure was frequently associated with increased lesion burden, although responses varied with particle characteristics, exposure conditions, and biological context. Importantly, administered doses varied substantially across studies, and several experimental models employed high exposure levels that are primarily informative for mechanistic proof-of-principle. Direct quantitative comparison with human internal MNP exposure remains difficult because exposure routes, particle characteristics, absorption, and dose metrics differ substantially between experimental and human studies. All in vivo findings are summarized in Table 2.

**Table 2:** Summary of endothelial and vascular effects of MNPs in vivo

Model system	Particle size & type	Exposure route	Exposure dose	Key endothelial and vascular findings	Ref.
Tg(kdrl: eGFP) zebrafish embryos	20 nm plastic particles (polymer not specified)	In medium for 48 h	2–8 mg/L	Impaired CVP development and vascular morphology; altered redox- and ECM-related pathways	Chen et al., [7]
C57BL/6 N mice (male)	1 μm, PS-COOH	Single i.v. injection	2.5 mg	Increased aortic *Il1b* and *Vcam1* expression	Vlacil et al., [48]
Swiss mice (male)	30 nm PS-COOH	Single i.v. injection (EBD co-injection)	1.5, 15, 30 mg/kg bw	Dose-dependent vascular leakage in brain, liver, spleen and lung; dermal microvascular leakage	Wei et al., [52]
BALB/c mice (male)	80 nm PS, PS-NH_2_ and PS-COOH	Repeated i.v. injections every other day for 14 days	PS: 5–20 mg/kg bw; PS-NH_2_/PS-COOH: 0.05–5 mg/kg bw	Structural vascular endothelial injury, inflammatory response, prothrombotic state associated with JAK1/STAT3/TF signaling	Wang et al., [51]
C57BL/6J mice (male)	42 nm PS	Daily oral gavage for 7 days	0.5–50 mg/kg bw	Increased BBB permeability	Shan et al., [45]
Wistar rats (male)	500 nm PS	Daily oral gavage for 90 days	0.5–50 mg/kg bw	Vascular injury, oxidative stress, inflammatory response, activation of pyroptosis-related signaling	Huo et al., [17]
C57BL/6J mice (male)	80 nm PS	Daily oral gavage for 12 weeks	80 mg/kg bw	Arterial wall thickening, increased collagen deposition, reduced arterial elasticity	Lv et al., [31]
ApoE^‒/‒^ mice (male)	100 nm PS	Oral gavage for 3 months	5 mg/kg and 10 mg/kg bw twice weekly	Increased atherosclerotic plaque burden and lipid deposition, reduced aortic blood flow velocity, increased plaque collagen content	Wen et al., [53]
ApoE^‒/‒^ mice (male)	50 nm PS	Daily oral gavage for 19 weeks + HFD	2.5 mg/kg bw, 25 mg/kg bw, 250 mg/kg bw	Increased arterial stiffness, atherosclerotic lesion burden, lipid accumulation, collagen deposition	Wang et al., [49]
LDLR^‒/‒^ mice (male and female)	1.1 μm PS	Daily oral gavage for 9 weeks + low-fat, low-cholesterol diet	10 mg/kg bw	Increased atherosclerotic lesion burden in male but not female mice, altered endothelial cell subpopulations in males	Lin et al., [24]
C57BL/6 mice (male)	1 μm PS	Drinking water for 18 weeks ± HFD	10 mg/L	Altered renal endothelial cell composition and activation of PI3K-Akt, MAPK, and IL-17 signaling pathways	Xu et al., [54]
ApoE^‒/‒^ mice (male)	5 μm PS	Drinking water for 12 weeks + HFD	1 and 10 µg/mL,	Increased atherosclerotic plaque burden, aortic oxidative stress, EndMT-associated changes	Yang et al., [55]
ApoE^‒/‒^ mice (male)	500 nm PS	Drinking water for 20 weeks	1 µg/mL	Increased lipid accumulation in the heart valve area; aortic transcriptomic changes involving immune activation, cell adhesion, and inflammatory pathways	Zhao et al., [60]
Sprague Dawley rats (male)	1–10 μm PET	Drinking water for 8 weeks	1, 10, 100 mg/L	PET accumulation in aortic endothelial glycocalyx loss, endothelial activation	Huo et al., [18]

## Clinical findings

The available human evidence addresses distinct aspects of MNP exposure and cardiovascular disease, ranging from detection in the circulation to accumulation in vascular tissues and associations with cardiovascular disease severity and clinical outcomes. These findings should be interpreted according to their respective level of evidence, as detection of MNPs in blood or vascular tissue demonstrates internal exposure but does not itself establish endothelial toxicity or a causal contribution to cardiovascular disease.

### Detection and systemic bioavailability

Leslie et al., provided the first evidence that plastic particles can be detected in human blood. Using a validated pyrolysis gas chromatography mass spectrometry (Py-GC/MS) workflow capable of identifying particles that are at least 700 nm in size, the authors analyzed whole blood from 22 healthy volunteers. Quantifiable concentrations above the limit of quantification were detected in 17 donors. PET was the most frequently detected polymer, followed by PS, PE, and PMMA. The mean summed concentration of quantifiable plastic particles was 1.6 µg/mL. These findings establish the presence of plastic particles in the human circulation and thus provide evidence of systemic bioavailability, but do not demonstrate vascular accumulation or endothelial effects [23].

### Vascular and atherosclerotic plaque accumulation

Several subsequent studies have provided evidence that MNPs are also detectable within human vascular tissues. One of the first studies to examine arterial MNPs in humans was conducted by Liu et al., who analyzed coronary arteries (*n* = 4), carotid arteries (*n* = 7), and aortic tissue (*n* = 6) using Py-GC/MS. MNPs were detected in all arterial samples. Concentrations were higher in coronary and carotid arteries than in aortic tissue. Coronary and carotid samples both originated from atherosclerotic lesions, whereas the aortic samples did not contain plaques, suggesting greater MNP accumulation in atherosclerotic vessels. The main polymers identified included PET, polyamide-66 (PA-66), polyvinyl chloride (PVC), and PE [26]. Although these findings suggest preferential MNP accumulation or retention in atherosclerotic tissue, the small sample size and cross-sectional design preclude conclusions regarding whether MNP deposition contributes to plaque development or occurs preferentially in already diseased vessels.

Cui et al., analyzed paired blood and carotid plaque samples from 20 patients undergoing carotid endarterectomy to compare circulating and tissue-associated MNPs. MNPs were detected in both compartments, with substantially higher concentrations in plaques compared with blood. In blood, PE was the predominant polymer, whereas plaque samples showed a broader distribution dominated by PP, PE, styrene-butadiene rubber (SBR), and PVC. Several polymers, such as PMMA, were frequently absent in blood but consistently detectable in plaques, indicating differential distribution between circulating and plaque-associated MNPs. When blood and plaque samples were directly compared within the same individuals, only weak correlations for total MNP burden were observed, suggesting that circulating levels only partially reflect plaque-associated deposition [9].

Further evidence for MNP accumulation in advanced atherosclerosis was reported by Massie et al., who examined femoral artery plaques from patients with peripheral arterial disease. Total MNP concentrations were approximately 80-fold higher in atherosclerotic femoral plaques than in non-atherosclerotic control arteries. Multiple polymers, including PE, PS, acrylonitrile butadiene styrene (ABS), SBR, PVC, PET, PMMA, PC, PA-66, and polyamide-6 (PA-6), were significantly enriched within plaque tissue. Patients with chronic limb-threatening ischemia exhibited markedly higher concentrations of PP and PU compared with patients with claudication [36].

Together, these tissue-based studies support an association between MNP burden and atherosclerotic vascular disease. However, preferential deposition or retention of MNPs within pre-existing lesions, rather than a causal contribution to atherogenesis, cannot be excluded.

### Associations with cardiovascular disease severity and clinical outcomes

Beyond MNP detection and vascular accumulation, observational studies have examined associations between MNP burden and cardiovascular disease severity and clinical outcomes. In patients presenting with acute chest pain, Yang et al., investigated circulating MNPs in relation to coronary artery disease. After predefined exclusions, 101 patients were included. MNPs were quantified in venous blood using Py-GC/MS and were detected in all participants, with mean concentrations of approximately 150 µg/g blood and peak levels exceeding 400 µg/g. The predominant polymers were PE, followed by PVC, PP, and PS. The anatomical complexity of coronary artery disease was assessed using the SYNTAX score, a standardized angiographic scoring system. Total circulating MNP levels, as well as PE, PVC, PS, and PP, correlated positively with SYNTAX scores, indicating an association between MNP burden and coronary lesion complexity. Total MNP concentrations were also positively correlated with the pro-inflammatory cytokines IL-6 and IL-12p70 [57]. These findings demonstrate an association between circulating MNP burden, coronary lesion complexity, and systemic inflammatory markers, but the cross-sectional design does not establish the direction or causality of these relationships.

The strongest clinical outcome data to date were reported by Marfella et al., who conducted a prospective, multicenter observational study in patients undergoing carotid endarterectomy for asymptomatic carotid artery disease. A total of 304 patients were enrolled, of whom 257 completed the follow-up. Excised carotid plaques were analyzed for MNPs using Py-GC/MS, complemented by stable isotope analysis and electron microscopy to confirm particle morphology and localization. MNPs were detected in a substantial proportion of carotid plaques, with PE identified in 58.4% of patients and PVC in 12.1%. Ultrastructural analyses revealed jagged-edged foreign particles within plaque macrophages and extracellular debris. Patients were followed for a mean duration of 33.7 months. During follow-up, the presence of MNPs within carotid atheroma was associated with a significantly increased risk of the composite clinical endpoint of myocardial infarction, stroke, or all-cause mortality. Patients with MNP-positive plaques exhibited a markedly higher event rate compared with those without detectable MNPs [32]. This study provides prospective observational evidence linking intraplaque MNP detection with adverse cardiovascular outcomes. However, its observational design does not establish that MNPs directly contributed to these events.

### Medical procedures as a potential source of intravascular MNP exposure

An additional consideration is the potential contribution of medical procedures to circulating MNP burden. Recent evidence suggests that medical procedures involving plastic-containing devices may represent an additional source of MNP exposure. In patients undergoing cardiac surgery, Yang et al., observed procedure-associated changes in the composition and size distribution of MNPs in pre- and postoperative blood samples, with several detected polymers corresponding to materials used in medical devices [56]. Liu et al., reported a marked increase in blood MNP concentrations following PCI compared with pre-procedural levels. The polymers detected after PCI corresponded to those identified in washing water from the interventional devices, supporting the devices as a potential source [27]. Similarly, Deng et al., found significantly higher blood MNP levels following hemodialysis, including several polymers that were not detected before treatment. Complementary simulation experiments further supported the dialysis system as a potential source [11]. However, the extent to which different medical procedures contribute to circulating MNP burden remains poorly characterized, and the persistence and potential biological consequences of such iatrogenic exposure are currently unknown.

**Fig. 3:**
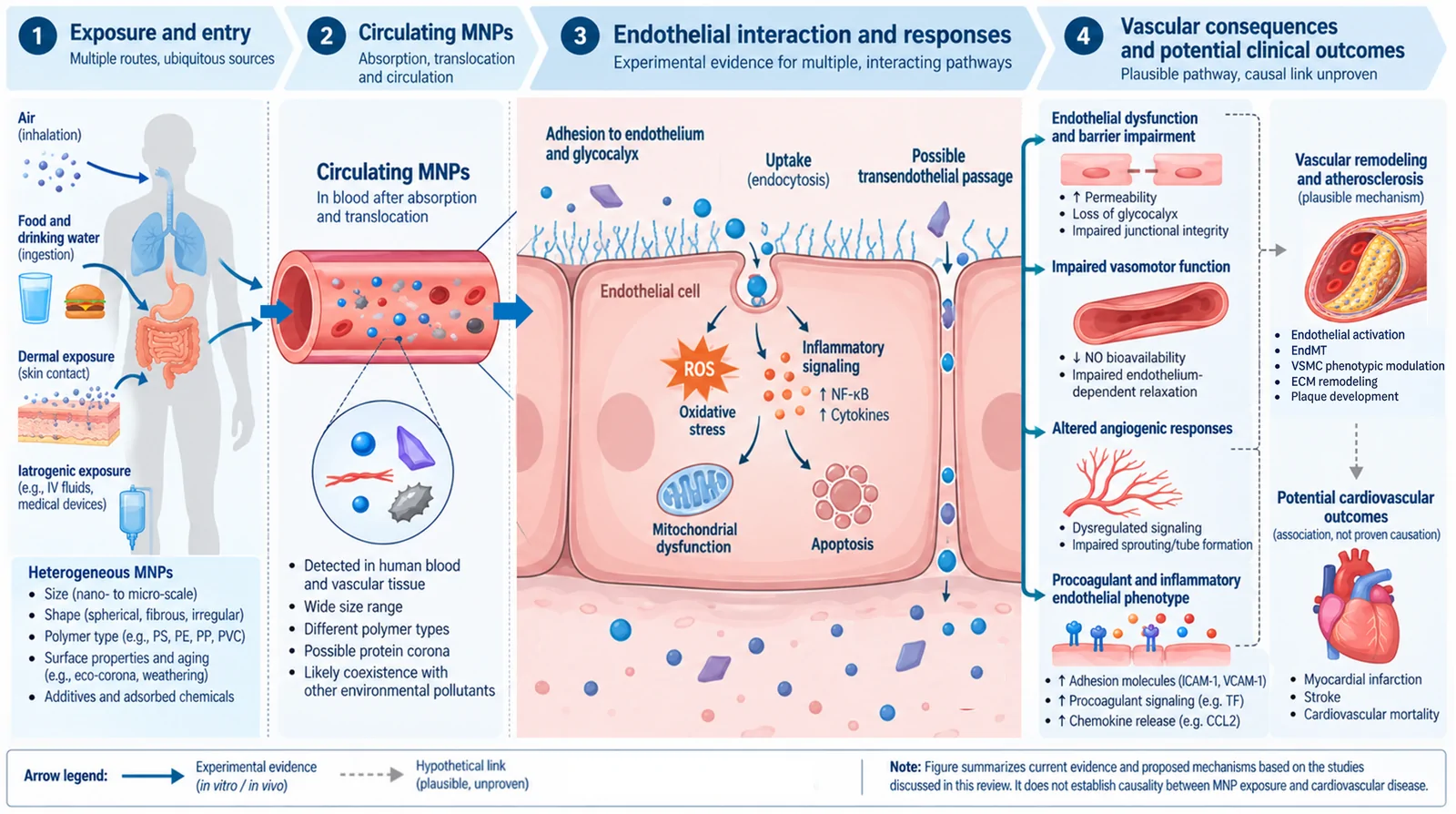
Proposed pathway linking MNP exposure to endothelial and vascular effects. MNPs may enter the body through inhalation, ingestion, dermal exposure or iatrogenic sources and subsequently reach the circulation. Once in the bloodstream, MNPs may interact with the vascular endothelium and glycocalyx, be taken up by endothelial cells, or potentially cross the endothelial barrier. Experimental studies have linked MNP exposure to oxidative stress, inflammatory signaling, mitochondrial dysfunction, and apoptosis in endothelial cells. These effects may contribute to impaired endothelial barrier integrity, altered vasomotor and angiogenic function, and a procoagulant and inflammatory endothelial phenotype. In vivo studies further suggest vascular remodeling and atherosclerotic changes following MNP exposure. Human studies have detected MNPs in blood and vascular tissues and reported associations with cardiovascular disease and adverse cardiovascular outcomes. However, whether endothelial dysfunction provides a causal link between MNP exposure and cardiovascular disease in humans remains unclear. Solid arrows indicate relationships supported by experimental evidence, whereas dashed arrows indicate links supported by observational findings but not established as causal relationships

Together, the available human studies demonstrate that MNPs can be detected in the circulation and vascular tissues and indicate associations between MNP burden, atherosclerotic disease severity, and adverse cardiovascular outcomes. However, the available evidence remains observational and does not establish that MNPs cause endothelial dysfunction or contribute directly to atherosclerosis. Moreover, direct clinical assessments of endothelial function are currently lacking. Thus, while the human findings support the vascular relevance of MNP exposure, the endothelial mechanisms identified in experimental studies remain largely unverified in humans. The current evidence and proposed pathways linking MNP exposure to endothelial and vascular effects are summarized in Fig. 3. All clinical studies meeting the predefined eligibility criteria are summarized in Table 3.

**Table 3:** Summary of human clinical observations related to MNP exposure

Patient cohort/ tissue	Quantified MNP burden (mean)	Detected polymers	Analytical method	Key findings	Ref.
Human coronary (*n* = 4) and carotid arteries (*n* = 7) containing atherosclerotic plaques, and non-atherosclerotic aortic tissue (*n* = 6)	Coronary: 156.5 µg/g tissue Carotid: 133.37 µg/g tissue Aorta: 76.26 µg/g tissue	PET, PA-66, PVC, PE	Py-GC/MS	MNPs detected in all arterial samples; higher concentrations in atherosclerotic coronary and carotid arteries than in non-plaque aortic tissue	Liu et al.,[26]
20 patients undergoing carotid endarterectomy; paired blood and plaque samples	75.2 µg/g in blood 432.9 µg/g in plaque	Blood: predominantly PE, followed by PVC and SBR Plaque: predominantly PP, PE, SBR, PVC	Py-GC/MS	Markedly higher MNP burden in plaques than in blood; weak correlation between circulating and plaque-associated MNPs; selective enrichment of specific polymers within atherosclerotic tissue	Cui et al., [9]
Patients with peripheral arterial disease; atherosclerotic femoral artery plaques compared with non-atherosclerotic control arteries	3234 µg/g tissue in femoral plaque 40.68 µg/g tissue in control arteries	PE, PS, ABS, SBR, PVC, PET, PMMA, PC, PA-66, PA-6, PP, PU	Py-GC/MS	Approximately 80-fold higher MNP concentrations in atherosclerotic plaques compared with control arteries; polymer-specific enrichment associated with disease severity	Massie et al., [36]
101 patients with coronary artery disease; venous blood samples analyzed and correlated with angiographic SYNTAX score	100.13 µg/g in blood in control group 161.65 µg/g in blood in ACS group	PE, PVC, PP, PS, PA-66	Py-GC/MS	Circulating MNP levels correlated positively with coronary lesion complexity (SYNTAX score) and systemic inflammatory markers (IL-6, IL-12p70)	Yang et al., [57]
304 patients undergoing carotid endarterectomy; carotid atherosclerotic plaques analyzed with prospective clinical follow-up	PE: 21.7 µg/g in plaque PVC: 5.2 µg/g in plaque	PE, PVC	Py-GC/MS; stable isotope analysis; electron microscopy	Presence of MNPs within carotid plaques is associated with a significantly increased risk of myocardial infarction, stroke, or all-cause mortality during follow-up	Marfella et al., [32]

## General limitations of studies with MNPs

Despite the rapidly expanding literature on MNP-mediated endothelial effects, several important limitations of the current evidence base must be considered.

Most mechanistic insights are derived from in vitro studies, which provide valuable information on endothelial stress responses and signaling pathways but cannot fully recapitulate the complexity of the vascular system in vivo. A broad range of endothelial models has been employed, with many studies relying on HUVECs as a widely accessible and well-characterized experimental system. HUVECs express key endothelial markers and inflammatory adhesion molecules and have therefore served as a robust endothelial model for studying general endothelial responses. However, their venous origin from the macrovasculature and from fetal tissue may limit direct extrapolation to adult, tissue-specific endothelial phenotypes [37]. Differences in barrier properties, inflammatory responsiveness, and regulatory signaling between macrovascular and microvascular endothelial cells may influence MNP-induced effects, particularly for processes such as barrier dysfunction, immune cell interaction, and angiogenic responses. Beyond model selection, a major limitation of in vitro studies relates to exposure conditions. Many reported effects occur at relatively high particle concentrations and often coincide with reduced cell viability. This complicates the distinction between specific endothelial signaling responses and secondary effects arising from generalized cellular stress. Moreover, direct comparison of experimental concentrations with internal MNP exposure in humans remains difficult because the concentrations applied in vitro cannot readily be related to the MNP burden that the vascular endothelium is exposed to in humans. Accordingly, findings obtained at high experimental concentrations should primarily be interpreted as mechanistic evidence of potential endothelial effects rather than as quantitative estimates of vascular risk under human exposure conditions. Another limitation is the predominance of acute exposure paradigms, whereas chronic, low-dose exposure has been investigated far less frequently, despite being more representative of continuous human environmental exposure. Consequently, adaptive responses and long-term endothelial remodeling processes remain insufficiently characterized.

The translational relevance of in vivo models requires careful contextualization. Early in vivo studies frequently relied on intravenous particle administration, which does not reflect environmentally relevant exposure routes and likely overestimates direct endothelial contact. Nevertheless, these models provided important proof-of-principle evidence that circulating MNPs can interact with the vascular endothelium and induce biological effects. At the same time, direct intravascular exposure may have clinical relevance in specific settings, as recent human studies suggest that medical procedures involving plastic-containing devices, including cardiac surgery, percutaneous coronary intervention (PCI), and hemodialysis, may introduce MNPs directly into the circulation. Thus, intravenous experimental models remain artificial with respect to conventional environmental exposure but may provide mechanistic insight into the consequences of direct vascular MNP exposure.

More recent studies have increasingly used oral exposure paradigms, including repeated gavage or drinking-water administration, which more closely approximate human exposure through ingestion. However, these approaches have important limitations. Oral gavage allows controlled and reproducible dosing but represents an artificial exposure route and may induce local mechanical irritation or stress responses in the upper gastrointestinal tract, which could indirectly influence inflammatory or vascular readouts. In contrast, drinking-water administration may more closely approximate continuous, voluntary oral exposure but provides less precise dose control because of individual variation in water consumption and potential particle sedimentation. Thus, current oral exposure models involve a trade-off between experimental dose control and environmental relevance.

Most experimental studies rely on pristine, sterile, and well-defined plastic particles under laboratory conditions. In environmental settings, however, MNPs undergo extensive physicochemical aging and rapidly acquire an eco-corona composed of adsorbed biomolecules, microorganisms, endotoxins, and organic pollutants [28]. This environmental corona is likely to substantially alter particle surface properties, cellular interactions, uptake mechanisms, and inflammatory potential. Because the vast majority of both in vitro and in vivo studies do not account for eco-corona formation, experimental findings obtained under controlled laboratory conditions may not fully reflect the biological behavior and toxicity of environmentally aged MNPs following human exposure. Closely related to this issue is the strong predominance of PS particles in experimental studies. PS is widely used because of its commercial availability, well-defined physicochemical properties, and experimental tractability. However, this practical advantage has resulted in a literature that is heavily skewed toward a single polymer, whereas other environmentally abundant plastics, such as PE, PP, or PVC, are comparatively underrepresented. The importance of particle composition and morphology is illustrated by recent findings from Martín-Pérez et al., who compared conventional spherical PS nanoparticles with biodegradable polylactic acid (PLA), irregular polytetrafluoroethylene (PTFE), and irregular PET nanoparticles derived from post-consumer plastic bottles and observed marked polymer-dependent differences in endothelial responses. Notably, PTFE and PET impaired endothelial migration under conditions in which PS showed no comparable effect, highlighting that findings obtained with standardized PS particles may not be readily generalizable to more environmentally representative particle types [34].

Beyond the limited representation of individual polymer types, another important limitation is that most experimental studies investigate single particle types in isolation. In contrast, human exposure to MNPs is inherently complex, involving simultaneous contact with mixtures of particles that differ in size, polymer composition, surface chemistry, and associated contaminants. Current experimental designs rarely address such cumulative or combinatorial exposure scenarios, leaving potential additive, synergistic, or antagonistic effects of heterogeneous particle mixtures largely unexplored. Furthermore, environmentally encountered MNPs may contain additives, plasticizers, and color pigments introduced during production and can occur as components of airborne particulate matter. These characteristics are rarely reproduced by the standardized particles used experimentally, further limiting their environmental relevance.

Sex represents an additional potential biological modifier that remains insufficiently addressed in current in vivo studies. Most experimental studies have relied predominantly or exclusively on male animals, limiting assessment of sex-specific vascular responses. Notably, Lin et al., reported differential atherosclerotic responses between male and female mice, indicating that sex may influence vascular susceptibility to MNP exposure [24]. Greater inclusion of both sexes will therefore be important for assessing the generalizability and translational relevance of experimental findings.

Exposure metrics also remain poorly standardized across experimental studies. MNP doses are reported using different mass-, number-, or volume-based units, while particle size and density vary substantially between studies. Consequently, identical mass concentrations may correspond to markedly different particle numbers and total surface areas, complicating direct comparison of dose-response relationships across experimental systems. This heterogeneity also complicates comparison between experimental exposure levels and internal MNP burdens reported in humans, making it difficult to establish exposure thresholds for potential endothelial effects under human exposure conditions.

Human biomonitoring and clinical studies introduce a different set of limitations. MNPs have been detected in blood and atherosclerotic plaques, while higher MNP burdens have been associated with greater atherosclerotic disease severity and adverse cardiovascular outcomes. However, the observational nature of these studies does not allow causal inference. It remains unclear whether MNPs actively contribute to endothelial dysfunction and vascular disease progression or whether they preferentially accumulate in individuals with pre-existing vascular pathology. Moreover, most available clinical studies have not directly assessed endothelial function, making mechanistic extrapolation from experimental endothelial models to human vascular disease necessarily indirect. An additional and often underappreciated limitation of current clinical studies is the potential influence of exposure-related confounders. Dietary intake represents an important route of human MNP exposure, and small interventional pilot studies suggest that the consumption of highly processed foods and extensive use of plastic food packaging are associated with increased MNP concentrations in human stool samples, reflecting higher oral intake [15]. At the same time, dietary patterns characterized by frequent consumption of highly processed or packaged foods are often linked to established cardiovascular risk factors, including metabolic dysregulation and chronic low-grade inflammation. In such settings, MNP burden may correlate with cardiovascular outcomes without representing an independent causal driver. Beyond dietary factors, MNP exposure is embedded within a broader environmental and socioeconomic context.

Individuals living in urban environments or working in occupations with higher particle exposure may simultaneously encounter MNPs and other airborne pollutants. This is particularly relevant for airborne MNPs, as they occur within complex particulate mixtures rather than as an isolated exposure. Urban populations may additionally be exposed to multiple environmental cardiovascular stressors, including air pollution, noise, artificial light, and heat [38]. Socioeconomic conditions may further influence both exposure patterns and cardiovascular risk. This overlap makes it difficult to determine whether associations observed between MNP burden and cardiovascular outcomes reflect an independent effect of MNPs or, at least in part, correlated environmental exposures. Interpretation of these associations is further complicated by analytical limitations in the detection of MNPs in biological samples.

Despite recent methodological advances, reliable identification and quantification of MNPs in blood and tissue remain technically challenging. Reported concentrations are influenced by sample collection and processing, contamination control, polymer identification, and method-specific limits of detection and quantification. Analytical platforms also differ in the size ranges and polymer types they can reliably detect, while results are reported across different biological matrices and using heterogeneous units. Absolute MNP concentrations reported across clinical studies should therefore not be interpreted as directly comparable measures of internal exposure. In particular, particles in the nanometer range are difficult to detect with currently available analytical techniques and may fall below method-specific detection limits, especially in complex human biofluids and tissues [1]. Consequently, current measurements may underestimate the internal MNP burden. This uncertainty complicates accurate exposure assessment and the interpretation of reported clinical associations.

## Discussion, summary, and conclusion

The available evidence supports the vascular endothelium as an important biological interface between circulating MNPs and the cardiovascular system. Across the experimental literature, oxidative stress, inflammatory activation, and impaired endothelial barrier integrity emerge as the most consistently reported endothelial responses to MNP exposure. Additional mechanisms, including mitochondrial dysfunction, inflammasome activation and pyroptosis, autophagic alterations, ferroptosis-like stress, cellular senescence, and EndMT, have been described but are supported by a smaller number of studies and should therefore not be considered equally established. In vivo studies extend these observations by demonstrating endothelial activation, altered barrier integrity, impaired angiogenesis and vasomotor function, and vascular remodeling in exposed animals, with individual studies additionally reporting glycocalyx disruption. However, not all vascular effects observed in vivo can be attributed specifically to endothelial injury. Human studies provide a further, but distinct, level of evidence: MNPs have been detected in the circulation and vascular tissues, including atherosclerotic plaques, and higher MNP burdens have been associated with greater atherosclerotic disease severity and adverse cardiovascular outcomes. Nevertheless, these observational findings do not establish that MNP-induced endothelial dysfunction mediates cardiovascular disease in humans. Taken together, the evidence therefore supports biological plausibility for endothelial involvement in MNP-associated vascular effects, while a direct causal pathway in humans remains to be established.

Importantly, endothelial interactions with MNPs may not be restricted to conventional molecular toxicological pathways. The particulate nature of MNPs raises the possibility of direct physical and biophysical interactions with the vascular endothelium. Experimental evidence indicates that particle properties can influence endothelial barrier integrity. Wei et al., demonstrated that anionic MNPs increased endothelial permeability and disrupted VE-cadherin [52]. More recently, PET MNP exposure was associated with loss of the endothelial glycocalyx and disruption of intercellular junctions, further suggesting that particle interactions at the endothelial surface may contribute to barrier dysfunction [18]. A recent preprint further suggests that photoaged and aggregated MNPs may affect endothelial mechanotransduction by increasing cellular stiffness and attenuating mechanosensitive calcium signaling, particularly through Piezo-type mechanosensitive ion channel component 1 (PIEZO1)-associated pathways [40]. Together, these observations raise the possibility that particle size, shape, surface properties, and aggregation state influence endothelial responses not only through molecular toxicity but also by altering the physical and mechanosensory properties of the endothelial interface. However, direct evidence that MNPs cause local endothelial compression, microvascular obstruction, or impaired tissue perfusion remains limited. These potential biophysical mechanisms therefore represent an important but still underexplored area for future investigation.

The potential retention of MNPs at the endothelial interface also has important implications for the interpretation of biomonitoring and tissue-distribution studies. Blood measurements provide only a snapshot of particles circulating at the time of sampling and may not capture MNPs that have already associated with or been retained within the vascular wall. Conversely, detection of MNPs by whole-tissue analysis cannot establish their precise localization within an organ, as particles detected after tissue digestion may reside within the vascular compartment rather than the tissue parenchyma.

Emerging experimental evidence suggests that vascular sequestration and subsequent redistribution may depend on particle size and route of administration. In rats, Zhang et al., observed rapid removal of 20 and 100 nm PS particles from the circulation following intravenous administration, accompanied by substantial accumulation in the thoracic and abdominal aorta. Notably, aortic levels of 20 nm particles increased over time, whereas those of 100 nm particles markedly declined, highlighting size-dependent differences in vascular distribution [59]. These considerations are particularly relevant for organs protected by specialized vascular barriers. MNPs have, for example, been detected in human brain tissue [3], but tissue-level detection alone cannot establish whether particles entered the parenchymal compartment or, depending on the route of exposure, how they reached the tissue. For studies investigating blood-borne distribution to the brain, spatially resolved localization is therefore required to distinguish particles retained within the cerebral microvasculature or associated with endothelial cells from particles that have crossed the BBB. More generally, distinguishing circulating, vascular-associated, and parenchymal MNPs will be important for understanding the relationship between biomonitoring measurements, tissue accumulation, and biologically relevant endothelial exposure.

Despite the growing mechanistic evidence, translating experimental findings to human cardiovascular risk remains challenging. Many in vitro studies employ MNP concentrations substantially higher than those that can currently be related to internal human exposure, and some endothelial effects occur under conditions accompanied by reduced cell viability. Similarly, animal studies frequently use exposure levels designed to establish biological effects rather than reproduce quantitatively defined human internal doses. These experiments are therefore particularly informative for identifying potential mechanisms and establishing proof-of-principle, but they cannot currently define exposure thresholds at which endothelial dysfunction would be expected in humans. This distinction is especially important because experimental dose metrics vary substantially between studies and human biomonitoring remains methodologically heterogeneous. At present, neither the vascular MNP burden experienced by the human endothelium nor its relationship to commonly used experimental concentrations can be defined with sufficient precision. Consequently, the available experimental literature should be interpreted as evidence of mechanistic plausibility rather than as a quantitative assessment of cardiovascular risk at current environmental exposure levels.

Exposure route represents another important determinant of how experimental findings should be interpreted. Intravenous administration bypasses epithelial absorption barriers and produces immediate vascular exposure and therefore does not reproduce conventional environmental exposure through ingestion or inhalation. However, direct intravascular exposure may have clinical relevance in specific iatrogenic settings, as increased circulating MNP burdens have been reported in association with cardiac surgery, PCI, and hemodialysis [11, 27, 56]. Intravenous animal models may therefore provide useful proof-of-principle evidence for particle-endothelium interactions following direct vascular exposure, while remaining unsuitable for quantitative extrapolation to conventional environmental exposure. Environmental and iatrogenic exposure should thus be considered distinct but potentially complementary contexts of endothelial MNP exposure.

More recent oral exposure models provide greater environmental relevance but introduce additional uncertainty regarding gastrointestinal absorption, systemic bioavailability, and the fraction of administered particles that ultimately reaches the vascular compartment. Recent findings from rainbow trout further suggest that MNP-associated signals can be detected in the circulation following non-intravenous exposure. Following seven-day waterborne exposure to biodegradable polylactic acid (PLA) microplastics, particle-associated signals were detected in peripheral blood, accompanied by pronounced transcriptional responses in macrophage-monocyte populations and altered predicted intercellular signaling involving endothelial cells [47]. Although these endothelial interactions were computationally inferred and require functional validation, the findings highlight the potential contribution of circulating immune cell-endothelial communication to vascular responses following systemic MNP exposure.

Several key knowledge gaps must be addressed before experimental endothelial mechanisms can be linked more directly to human cardiovascular disease. Experimental studies should increasingly prioritize chronic, low-dose exposure paradigms and better reproduce the physicochemical complexity of environmentally encountered particles, including aged MNPs, heterogeneous polymer mixtures, irregular morphologies, and environmentally acquired coronas. Greater attention should also be given to endothelial heterogeneity, as endothelial phenotypes differ substantially between vascular beds and may respond differently to MNP exposure. Standardization of dose reporting, particle characterization, and analytical approaches will be essential to improve comparability across studies and to relate experimental exposure more meaningfully to human internal burdens. The physical interaction of particles with the endothelial surface, glycocalyx, junctional complexes, and mechanosensory pathways also warrants further investigation, particularly under physiological flow conditions. In vivo studies should incorporate both sexes and distinguish direct endothelial effects from secondary vascular responses wherever possible. In parallel, human studies require rigorous contamination control, standardized MNP detection and quantification, spatially resolved tissue analyses, and improved assessment of relevant environmental and lifestyle confounders. Most importantly, prospective studies combining individual MNP exposure or burden with direct measures of endothelial function would help determine whether the endothelial mechanisms identified experimentally are also operative in humans.

In conclusion, current evidence identifies the vascular endothelium as a plausible and potentially important interface for the cardiovascular effects of circulating MNPs. Experimental studies demonstrate that MNP exposure can disrupt endothelial homeostasis through oxidative and inflammatory signaling, barrier impairment, and additional context-dependent cellular pathways, while emerging evidence suggests that the physical properties of the particles themselves may also influence endothelial interactions. Human studies confirm systemic and vascular exposure and report associations between MNP burden and cardiovascular disease, but they do not yet establish MNP-induced endothelial dysfunction as a causal mediator of these associations. Resolving this gap will require environmentally and physiologically relevant experimental models, improved characterization of vascular particle localization and exposure, standardized analytical methods, and direct assessment of endothelial function in human studies. Such approaches will be essential to determine whether the endothelial effects established experimentally translate into clinically relevant vascular consequences under human exposure conditions.

## Supplementary Information

Below is the link to the electronic supplementary material.

Supplementary Material 1

## Data Availability

All data generated or analyzed during this study are included in this published article and its supplementary information files.

## Declarations

### Generative AI and AI-assisted technologies

ChatGPT (OpenAI) was used to assist in the generation of Fig. 3. The authors reviewed and approved the final figure and remain fully responsible for its content.
