# Supplementary material for: Micro- and nanoplastics at the vascular interface: experimental insights into endothelial pathways and clinical evidence
Source: Part Fibre Toxicol. 2026 Nov 11;23:45. doi: 10.1186/s12989-026-00714-4 (PMC13756977; doi:10.1186/s12989-026-00714-4)
Supplement: Supplementary file 1 [file 12989_2026_714_MOESM1_ESM.docx]

**Abbreviations**

| ABS | acrylonitrile butadiene styrene |
| --- | --- |
| ACS | acute coronary syndrome |
| AGGF1 | angiogenic factor with G-patch and FHA domains 1 |
| Akt | protein kinase B |
| ApoE^‒/‒^ mice | apolipoprotein E-deficient mice |
| aPTT | activated partial thromboplastin time |
| ASC | apoptosis-associated speck-like protein containing a caspase recruitment domain |
| ATP | adenosine triphosphate |
| Bax | Bcl-2-associated X protein |
| BBB | blood-brain barrier |
| Bcl-2 | B-cell lymphoma 2 |
| bEND.3 | brain endothelial cells |
| BfR | German Federal Institute for Risk Assessment |
| BMP-2 | bone morphogenetic protein 2 |
| BMP-6 | bone morphogenetic protein 6 |
| Bw | body weight |
| CCL2 | C-C motif chemokine ligand-2 |
| CD31 | cluster of differentiation 31 |
| CD36 | cluster of differentiation 36 |
| CVP | caudal vein plexus |
| EC | endothelial cell |
| ECM | extracellular matrix |
| EndMT | endothelial-to-mesenchymal transition |
| eNOS | endothelial nitric oxide synthase |
| ERK1/2 | extracellular signal-regulated kinases 1 and 2 |
| Fabp4 | fatty acid-binding protein 4 |
| FAK | focal adhesion kinase |
| FHA | forkhead-associated domain |
| FTH1 | ferritin |
| GPX4 | glutathione peroxidase 4 |
| GSDMD | gasdermin D |
| GSH | glutathione |
| HAEC | human aortic endothelial cell |
| H_2_O_2_ | hydrogen peroxide |
| hCMEC/D3 | human brain microvascular endothelial cell |
| HFD | high-fat diet |
| HMEC-1 | human microvascular endothelial cell line |
| HO-1 | heme oxygenase-1 |
| hpf | hours post-fertilization |
| HRMEC | human primary retinal microvascular endothelial cell |
| HSP70/90 | heat shock proteins 70 and 90 |
| HUVEC | human umbilical vein endothelial cell |
| ICAM-1 | intercellular adhesion molecule-1 |
| IL-10 | interleukin~~e~~ 10 |
| IL-18 | interleukin 18 |
| IL-1β | interleukin 1β |
| IL-6 | interleukin 6 |
| JAK1 | janus kinase 1 |
| LDH | lactate dehydrogenase |
| LAMP2 | lysosome-associated membrane protein 2 |
| LC3 | microtubule-associated protein 1A/1B-light chain 3 |
| LC3-II | microtubule-associated protein 1 light chain 3-II |
| LDLR^‒/‒^ mice | low-density lipoprotein receptor-deficient mice |
| MAPK | mitogen-activated protein kinase |
| MDA | malondialdehyde |
| MLKL | mixed lineage kinase domain-like protein |
| MMP-9 | matrix metalloproteinase-9 |
| MNP/MNPs | micro- and nanoplastic(s) |
| MyEnd | murine myocardial microvascular endothelial cell |
| NAC | N-acetylcysteine |
| NF-κB | nuclear factor kappa-light-chain-enhancer of activated B cells |
| NLRP3 | NLR family pyrin domain-containing 3 |
| NO | nitric oxide |
| NOS3 | endothelial nitric oxide synthase |
| PA-6 | polyamide-6 |
| PA-66 | polyamide-66 |
| PAR1 | protease-activated receptor-1 |
| PC | polycarbonate |
| PCAEC | primary porcine coronary artery endothelial cell |
| PCI | percutaneous coronary intervention |
| PE | polyethylene |
| PECAM-1 | platelet endothelial cell adhesion molecule-1 |
| PET | polyethylene terephthalate |
| PI3K | phosphoinositide 3-kinase |
| PIEZO1 | piezo-type mechanosensitive ion channel component 1 |
| PLA | polylactic acid |
| PLCγ1 | phospholipase C gamma 1 |
| PM | particulate matter |
| PMMA | polymethyl methacrylate |
| PP | polypropylene |
| PRDX1 | oxidative stress-related enzyme peroxiredoxin 1 |
| PS | polystyrene |
| PT | prothrombin time |
| PTFE | polytetrafluoroethylene |
| PU | polyurethane |
| PVC | polyvinyl chloride |
| Py-GC/MS | pyrolysis gas chromatography mass spectrometry |
| RIPK3 | receptor-interacting protein kinase 3 |
| ROS | reactive oxygen species |
| Sa-β-gal | senescence-associated β-galactosidase activity |
| SBR | styrene-butadiene rubber |
| SDC1 | syndecan-1 |
| SGLT2 | sodium-glucose co-transporter 2 |
| SMAD | suppressor of mothers against decapentaplegic homolog |
| SMC | smooth muscle cell |
| SOD | superoxide dismutase |
| SRC | proto-oncogene tyrosine-protein kinase Src |
| STAT3 | signal transducer and activator of transcription 3 |
| TEER | transendothelial electrical resistance |
| TF | tissue factor |
| TNF-α | tumor necrosis factor alpha |
| UN | United Nations |
| VCAM-1 | vascular cell adhesion molecule-1 |
| VE-cadherin | vascular endothelial cadherin |
| VEGF | vascular endothelial growth factor |
| ZO-1 | zonula occludens-1 |
| α-SMA | α-smooth muscle actin |
